# Soluble mediators in the function of the epidermal-immune-neuro unit in the skin

**DOI:** 10.3389/fimmu.2022.1003970

**Published:** 2022-10-18

**Authors:** Ewa Oleszycka, Kamila Kwiecien, Patrycja Kwiecinska, Agnieszka Morytko, Natalia Pocalun, Michelle Camacho, Piotr Brzoza, Brian A. Zabel, Joanna Cichy

**Affiliations:** ^1^ Department of Immunology, Faculty of Biochemistry, Biophysics and Biotechnology, Jagiellonian University, Kraków, Poland; ^2^ Palo Alto Veterans Institute for Research, VA Palo Alto Health Care System, Palo Alto, CA, United States

**Keywords:** Skin, innate immunity, sensory neurons, cytokine, chemokine, neuropeptide, antimicrobial peptide, protease

## Abstract

Skin is the largest, environmentally exposed (barrier) organ, capable of integrating various signals into effective defensive responses. The functional significance of interactions among the epidermis and the immune and nervous systems in regulating and maintaining skin barrier function is only now becoming recognized in relation to skin pathophysiology. This review focuses on newly described pathways that involve soluble mediator-mediated crosstalk between these compartments. Dysregulation of these connections can lead to chronic inflammatory diseases and/or pathologic conditions associated with chronic pain or itch.

## Introduction

The skin relies on several major cellular networks to detect and react to environmental challenges such as microbial threats or physical/chemical insults. While the outermost skin layer -the epidermis - is strategically positioned to cope with various environments, its defensive actions are enhanced by the cutaneous immune system and skin-innervating sensory afferents. The immune and nervous systems provide complementary strategies to defend the barrier. The involvement of each system in the overall skin-protective response is largely dictated by skin anatomy and by the time scale of its operational capacity. For example, the nervous system requires a millisecond time frame to convey information about barrier challenge from the skin to the brain. These signals are typically initiated at the level of the sensory neuron endings in the skin and transmitted to neuronal cell bodies located in sensory ganglia, such as dorsal root ganglia (DRG), and further to the spinal cord. The signals ascend to the brain, where they are perceived as pain, itch or other sensations. On the other hand, the immune system operates on a time scale of minutes-to-days, and largely employs migratory leukocytes that travel to different cutaneous sites to exert protective activities. Importantly, epidermal keratinocytes and immune cells such as skin resident mast cells or dendritic cells (DCs), are located in close proximity to cutaneous axonal terminals and thus are anatomically poised to form regulatory circuits in the skin. This review aims to explore soluble protein/peptide-based mediators that causally link the epidermis, the immune system and the sensory nervous system and highlight how the resultant triad operates as a functional unit in skin protection. We also discuss the maladaptive responses of this “skin functional connectome” that can contribute to chronic inflammatory conditions and/or persistent itch or pain.

In the first three sections we will provide a layout of the epidermis, the immune system and the nervous system in the skin, highlighting the shared and unique functions of each compartment in skin protection and pathology.

### The epidermis

**Figure 1:**
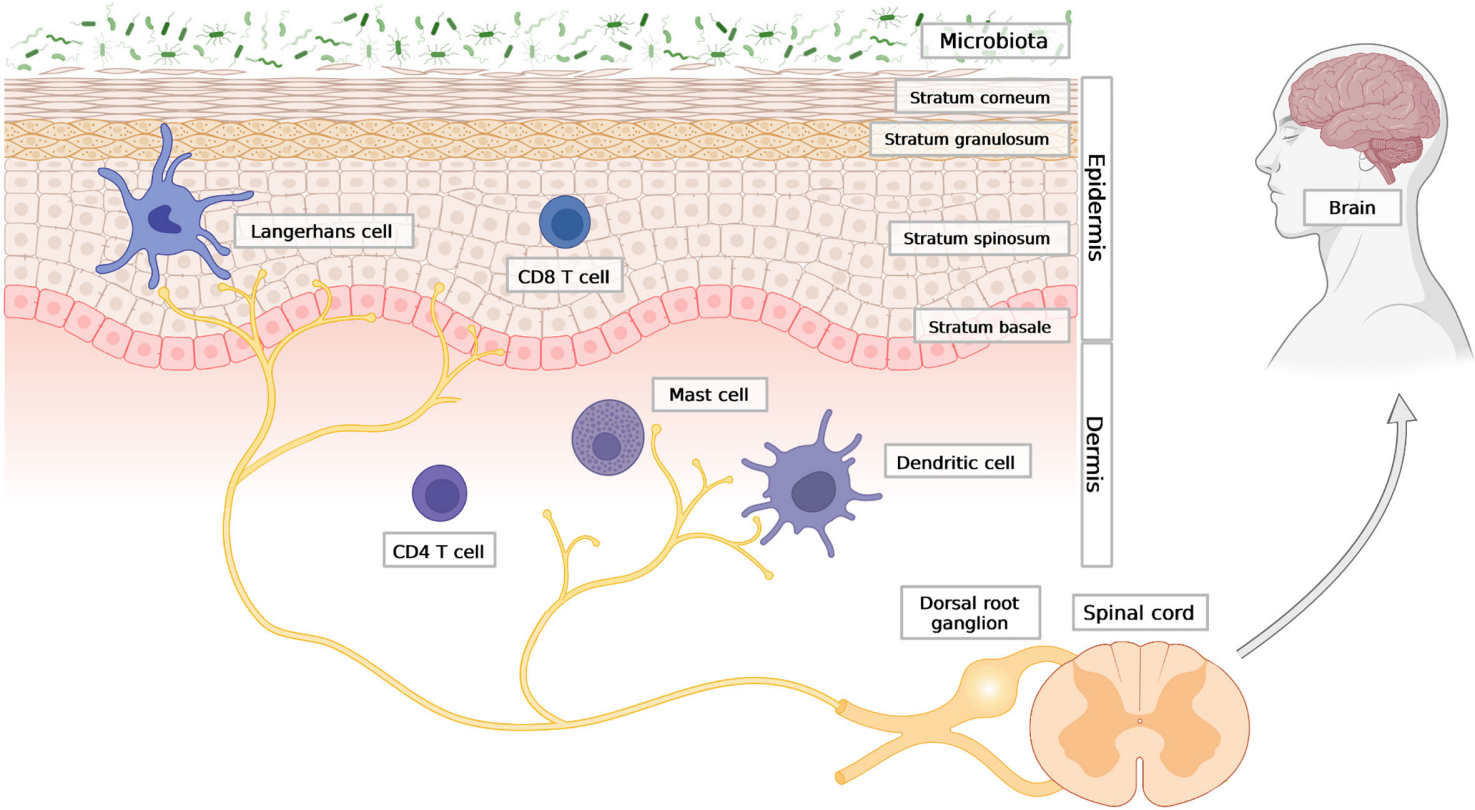
The outline of skin architecture with main points of anatomical contact between the epidermis, the immune and the neuronal networks. Three main skin compartments are shown: the stratified epidermis; representative cellular components of innate and adaptive immunity; the nervous system, with afferent endings in close contact with Langerhans cells (LCs) in the epidermis, as well as dermal DC and mast cells in the dermis. Created with BioRender.com.

The skin has a stratified structure that includes three main layers: (i) the top, keratinocyte-dominated layer, termed epidermis; (ii) the middle layer composed mostly of fibroblasts, termed dermis; and (iii) the bottom layer consisting primarily of adipocytes, termed hypodermis. Keratinocytes are the most abundant cell type in the epidermis and are positioned directly at the interface with the external environment, where exposure to toxins, allergens and other insults is frequent (1). Skin microbial communities, collectively known as skin microbiota, can contain bona fide invasive (pathogenic) microorganisms, and are also a constant threat to skin homeostasis (2, 3). Keratinocytes that guard the epithelial skin barrier are arranged in layers that differ in morphology and function (**Figure 1**). The lowest layer of the epidermis – the stratum basale - is separated from the dermis by a basement membrane and composed of undifferentiated keratinocytes that possess high proliferative potential and the ability to renew all epidermal layers (4). The basal keratinocyte monolayer constantly delivers new cells that differentiate to create upper epidermal strata comprising the stratum spinosum, stratum granulosum and stratum corneum. The stratum spinosum is built of several layers of keratinocytes that gradually acquire new features as they differentiate and migrate outward to create the stratum granulosum. The granular layer contains keratinocytes filled with lamellar bodies – secretory granules enriched in lipids and preformed antimicrobial proteins and peptides (AMPs). Lamellar bodies provide the cell-crosslinking extracellular matrix for the flattened, enucleated, terminally differentiated keratinocytes of the stratum corneum (5). Keratinocytes from this layer are constantly shed during the process of desquamation, which is mediated by a family of serine proteases known as kallikreins (KLKs) (6, 7). The stratum corneum together with the tight junctions that fasten adjacent keratinocytes in the stratum granulosum forms an interdependent permeability and antimicrobial barrier (4). This structure controls transepidermal water loss and largely relies on AMPs to prevent microbe penetration through the barrier (8).

Due to their skin localization and expression of pattern-recognition receptors (PRRs) such as Toll-like receptors (TLRs), keratinocytes are perfectly positioned to initiate the cutaneous responses to microbial challenges (9). The ability of keratinocytes to release soluble mediators such as AMPs (e.g. cathelicidin, β-defensins, SLPI, GPR15L), cytokines (e.g. IL-1, TSLP, IL-33), chemokines (e.g. CXCL8) or proteases (e.g. KLKs) that can stimulate immune and/or neuronal cues (10–16), is in-line with the functional significance of keratinocytes in cuing skin-shielding pathways. However, since the cutaneous immune system or the somatosensory nervous system can also sense environmental insults (17, 18), keratinocytes may function to enhance or regulate downstream events that are first initiated by immune cells and/or neurons. These multiple cutaneous detection systems that consist of keratinocytes, skin resident immune cells or sensory neuronal fibers likely enable broad recognition of different threats and subsequent initiation of context-dependent responses that result in different protective outcomes.

Given that skin inflammation, as well as pain and/or itch are all skin defensive mechanisms, these processes often occur in parallel to optimize host response, but if dysregulated, can result in chronic inflammation and/or behavioral abnormalities such as uncontrolled scratching. The combined action of the epidermal-immuno-neuronal skin components often starts with keratinocyte-specific factors like antimicrobial peptides LL-37 or β-defensins, or cytokines like TSLP or IL-33, and culminates in skin protection, or if dysregulated, in pathology. The AMPs are produced and released from keratinocytes upon stress or damage and stimulate immune cells, such as mast cells to release pro-inflammatory cytokines, and/or pain- or itch-inducing mediators, which in turn activate sensory neurons (19, 20). Some AMPs, including β-defensins can also directly activate neurons (15). Likewise, following skin challenge, stressed keratinocytes release TSLP or IL-33 that can directly stimulate sensory fibers to evoke itch while also activating a variety of innate and adaptive immune cells, including mast cells and Th2 cells (13).

Collectively, these examples illustrate that the epidermal component of the epidermal-immune-neural functional unit is key to the initiation and propagation of immune and behavioral events that maintain and regulate skin barrier properties.

### The immune system

Immune cells of both innate and adaptive immunity can be found in every layer of the skin, but they mainly reside in the epidermis and dermis, where an encounter with skin surface microbes that breached the barrier is most likely to take place. The first line of immune defence is formed by antigen-presenting cells, which include Langerhans cells (LCs) and dermal dendritic cells (dDCs). LCs are mainly confined to the epidermis (**Figure 1**). The range of antigens that LCs are able to capture may extend to the stratum corneum and the basal layer (21). Initially LC were regarded as antigen presenting cells which can sense local inflammatory response and activate T cells (22, 23). However subsequently, it has been discovered that LCs are predisposed to tolerance induction and immunosupressive cutaneous reactions (24, 25). LCs localize close to nerve endings and also have an important role in maintaining innervation of the epidermis, since long-term depletion of LCs in the skin causes loss of neurons in the epidermis (26). Deeper into the skin, there are dDCs, including CD301b+ dDC, which similar to LCs, are often found in close proximity to nerve fibers. A parallel anatomic association exists for mast cells and neurons (27–29), (**Figure 1**). Mast cells are commonly found in the dermis (30) but rely on upper skin layers, as their maturation requires stem cell factor (SCF) produced by keratinocytes. Moreover, the frequency of mast cells in the skin is dependent on microbiota and pathogen-sensing. Germ-free mice have fewer mast cells in the dermis, which can be restored to near normal levels following reconstitution of bacteria (31). Apart from myeloid cells, the skin is also patrolled by memory T cells. T cells are abundant in both the epidermis (mostly cytotoxic CD8+ T cells) and dermis (mostly CD4+ T helper cells) (4), (**Figure 1**).

When the skin barrier is compromised or damaged, different subsets of circulating leukocytes infiltrate the skin, adding diversity and numbers to the skin immune landscape. Leukocytes migrate to the skin from dermal blood vessels (4). This requires effective communication between the epidermal, dermal and the immune cell networks, and is largely mediated by cytokines and chemokines, or in case of neurons by neuropeptides (see below). The barrier breach is an important decisive step for the development of specific types of long-term adaptive immunity, such as Th17 (type 3) immune responses against extracellular bacteria and fungi, or type 2 immune responses against parasitic worms and allergens. Whereas bacteria or fungi express conserved features that can be detected by PRRs on a variety of cutaneous cells, including immune cells, allergens do not derive from microbes and can be recognized based on their functional characteristics such as protease activity (18). A breach in the barrier is typically met with full-blown defensive responses that can result in different outcomes, such as proinflammatory Th17 immunity, or tissue repair-promoting Th2 immunity. Under the first scenario, cytokines like IL-17 produced by Th17 cells, γδ T cells, and/or group 3 innate lymphoid cells (ILC3) play a key role in inflammatory events. Under the second scenario, cytokines such as IL-4 or IL-13 produced by Th2 cells and/or group 2 innate lymphoid cells (ILC2), orchestrate tissue healing processes (18, 32).

Inflammatory changes initiated as a result of barrier damage can be first seen in the DC compartment, which is well-equipped - to interpret skin insults and initiate immune responses. For example, activated LCs migrate from the epidermis to the nearest lymph node (33) where they can secrete IL-23 and activate IL-17-dependent inflammation, which can occur in psoriasis (34). There are also additional subsets of DCs, such as plasmacytoid DCs (pDCs), which are not present in the skin under homeostatic conditions, but can be detected during skin inflammatory responses. PDCs specialize in restricting viral infection *via* rapid secretion of type I interferons (IFNI), and are implicated in psoriasis pathology (20, 35).

Following breakdown of the epidermal barrier, as the existing cutaneous response fails to restrict microbes, different populations of leukocytes are recruited from the blood to intercept and fight intruders. Among the first responders are neutrophils. Although large numbers of neutrophils can infiltrate the skin under both acute or chronic inflammatory conditions, the function of these granulocytes in chronic inflammatory skin diseases like psoriasis remains obscure. In addition to their association with inflammation, recent reports indicate that neutrophils also contribute to pain and itch (16, 36).

Whereas acute pain or itch that often accompany acute inflammation are important components of the skin protective function, chronic pain and itch are both debilitating conditions that can last for months or years. The cellular and molecular factors responsible for the transition from acute to chronic pain or itch remains to be determined. However, recent findings point to the importance of a normal, robust neutrophil-dominant inflammatory response during the acute stage in avoiding chronic pain. Transcriptome-wide analysis of human individuals suffering from chronic lower back pain revealed that if sufficient inflammatory processes are either not initiated or are pharmacologically disrupted, this increases the risk of developing chronic pain (36). Human transcriptomics results indicated that transient neutrophil-mediated up-regulation of inflammatory responses was protective against the transition to chronic pain. In line with these findings, in experimental complete Freund’s adjuvant-stimulated inflammatory hind paw injury, elevated neutrophil frequency during the acute pain stage protected against chronic pain development (36).

Although skin-infiltrating neutrophils are typically a hallmark of Th17-associated pathologies such as psoriasis, these granulocytes were recently reported to play a role as early mediators of chronic itch in the settings of atopic dermatitis (AD), a type 2-like chronic immune disorder. Itch is a prevalent feature of AD, recognized as part of its overall pathology. Using an experimental model of AD-like skin inflammation induced by vitamin D analog MC903, neutrophils were found to be required for development of chronic itch, as their depletion significantly reduced itch-evoked scratching (16).

However, itch is thought to represent a behavioral extension of type 2 immunity, and is typically associated with activation of mast cells, eosinophils and basophils. As already mentioned, this type of immunity largely controls host responses to extracellular parasites or allergens, and the itch-scratch cycle helps to expel intruders or irritants. Upon activation, these granulated cells deploy a variety of itch-inducing molecules (pruritogens), such as histamine. Histamine is released from mast cells in response to antigen-dependent cross-linking of IgE bound to FcεRI on surface of mast cells (37). Although targeting IgE and histamine signaling pathways have been the dominant strategy against pruritus, individuals suffering from different forms of chronic itch, including AD, have been largely refractory to anti-histamines, suggesting the involvement of alternative mechanisms (38–40). Recent advances demonstrated another key pathway underlying mast cell-dependent chronic pruritus. This pathway engages a different receptor on mast cells, MrgprB2 (Mas-related G-protein coupled receptor B2), instead of FcεRI. Mast cell activation *via* MrgprB2 leads to excitation of itch sensory neurons (41).

Taken together, the skin barrier defense provided by the immune compartment enables; detection of a wide range of pathogens, acute inflammatory responses to destroy invading microorganisms, and the opportunity for life-long adaptive immunity. Either innate leukocytes or lymphocytes form functional circuits with the nervous system to evoke pain and/or itch, which can be protective or lead to debilitating conditions if the pathway is dysregulated.

### The nervous system

The peripheral nervous system, which innervates the skin, consists of the autonomic nervous system that regulates skin physiology without conscious control (e.g. sweating) and the somatosensory nervous system that helps to quickly alert the body of external threats (13). The functional communication between the autonomic nervous system, the epidermis and immune compartment remains largely unexplored. In contrast, sensory neurons have recently been reported to directly decode pathogens and orchestrate specific immune responses at barrier surfaces *via* release of neuropeptides (42–45). These discoveries have led to a deeper appreciation of the importance of the somarosensory nervous system in skin defense and regulatory strategies.

The main role of the cutaneous somatosensory nervous system is to interpret the environment and initiate rapid protective behavioral responses, such as withdrawal to avoid skin damage and alleviate pain sensation, or scratching to expel irritants. Sensory neurons that innervate the skin have long axons originating from cell bodies that are clustered in regions known as the dorsal root ganglia (DRG), located close to the spinal cord, or the trigeminal ganglia located near the base of the skull. Whereas the trigeminal ganglion houses nerves that innervate the skin and organs of the head and face, sensory neurons in the DRG project peripheral afferent fibers to the skin and organs in the rest of the body (39, 46).

DRG neurons are pseudounipolar, with bifurcated axons: one axon extends to the skin or other tissues to receive outside signals, and the other synapses with a network of neurons in the spinal cord to relay sensory information to the brain for processing (47, 48), (**Figure 1**). Sensory neurons are morphologically and functionally diverse, and can be classified based on their axonal diameter, which is directly proportional to the degree of myelination, and to conduction speed. They are classified as follows: i) heavily myelinated and fast-conducting Aβ fibers; ii) slightly myelinated and medium-conducting Aδ fibers; and iii) unmyelinated, slow-conducting C fibers (46, 49). Different types of external stimuli are recorded and processed differently, with fast noxious (pain-inducing) stimuli, and slower pain and itch sensations being transmitted by Aδ and C fibers, respectively (50, 51). More recent approaches, such transcriptomic profiling, further divide these neurons into several subtypes based on their molecular signatures, such as expression of sensory receptors, ion channels and/or neuropeptides (52).

When specific signals act on terminal axons in the skin, this triggers the opening of nonspecific cation channels, including transient receptor potential V1 (TRPV1), and/or transient receptor potential A1 (TRPA1), resulting in membrane depolarization and subsequent activation of an action potential by voltage-gated ion channels, such as Na_v_1.7, Na_v_1.8 and Na_v_1.9 (53). An action potential travels to the DRG and finally to the spinal cord and the brain. Sensory neurons are also known to secrete and communicate *via* neuropeptides, such as calcitonin gene-related peptide (CGRP) and substance P (SP), TAFA4, pituitary adenylate cyclase-activating polypeptide (PACAP) or other neurotransmitters, such as glutamate (26, 43, 54, 55).

The importance of the nervous system in controlling the epidermal and immune compartments in the skin has been known for many years, as surgical or pharmacological denervation or nerve injury can significantly impact keratinocyte biology and cutaneous inflammation (29, 56). For example, keratinocytes were reported to increase their proliferation rate under the influence of CGRP produced by sensory neurons, suggesting that under homeostatic conditions skin thickness might be regulated by the nervous system (56). Among the best characterized sensory afferents in the skin are TRPV1+ neurons, which can impact immune cells *via* release of vesicle-stored neuropeptides, such as CGRP or SP (43). These neurons not only sense noxious or pruritic stimuli, resulting in painful or itchy sensations, but also link pathogen recognition and allergen sensing with DC or mast cell activation, and the subsequent development of Th17 or Th2 immune responses (28, 29, 43–45).

Due to the complexity and multidirectional interaction among the epidermis, the immune system and the nervous system, it has been difficult to dissect whether keratinocytes or immune cells are required to trigger neuronal cell-mediated sensory signals following cutaneous microbial infection or tissue damage. Alternatively, recent studies have shown that it is possible to initiate inflammation in the skin *via* sensory nerve activation alone. Using an optogenetic model in which TRPV1+ nociceptive neurons were activated by a blue light due to forced expression of the light-gated cation channel rhodopsin in these fibers, TRPV1+ neuron activation in the absence of microbial infection or damage-associated molecules was sufficient to trigger a Th17 immune response (43).

Moreover, the unique anatomical distribution of sensory afferents that connect different parts of the body, and their mode of action *via* reflex arcs, suggested that the nervous system might help to spread local activation signals and ultimately neuron-mediated events from one region of the skin to others. In a local axonal reflex arc, sensory neuronal signals initiated at the site of the original skin challenge and traveling towards the spinal cord back-propagate from axonal branch points, redirecting the signal back to axonal terminals in adjacent skin (13, 57). An interesting example of such neuronal involvement beyond the site of its original activation is the anticipatory Th17 immune response against the fungus *C. albicans* (43). Stimulation of sensory TRPV1+ nociceptive neurons by *C. albicans* at a single skin location resulted in the activation of nerve terminals in adjacent, uninvolved skin sites. This led to augmented, Th17 immune responses in these neighboring skin areas accompanied by lower fungal burden (43). A similar reflex-arc mediated phenomenon was also found to enlarge the size of local, imiquimod (IMQ)-induced skin alterations in a psoriasis-like dermatitis model. These skin changes manifested in an enhanced, pathology-associated loss of water through the compromised epidermal barrier (58).

Sensory TRPV1+ neurons are not only involved in GPCR-mediated Th17 immunity, but appear equally important for allergic Th2 immunity *via* release of SP (28, 45). Moreover, mounting evidence suggest that cutaneous neuro-immune pathways are also driven by other types of sensory neurons that do not express TRPV1. These include: (i) C-fibers that express Gαi-interacting protein (GINIP) and release the neuropeptide TAFA4, or (ii) epidermis innervating neurons, identified based on the expression of Mas-related G-protein-coupled receptor D (MrgprD). The TAFA4 producing GINIP+ sensory neurons were recently found to play a crucial role in tissue repair in a model of sunburn-like (UV)-induced skin damage by promoting IL-10 production by dermal macrophages (54). On the other hand, MrgprD-expressing neurons are dependent on LCs, and maintain skin homeostasis by suppressing mast cells *via* glutamate (26).

These findings suggest that the skin neuronal networks act cooperatively with the immune system to provide optimal cutaneous defense and coordinate inflammatory responses. Somatosensory neurons can detect and rapidly trigger behavioral responses (e.g. scratching to remove an allergen irritant) as well as transmit signals to uninvolved skin areas and interact with the nerve-associated DCs or mast cells to regulate immune cell numbers and activation in the skin.

## Soluble mediators in the skin epidermal-immuno-neural connections

### Cytokines and chemokines

**Figure 2:**
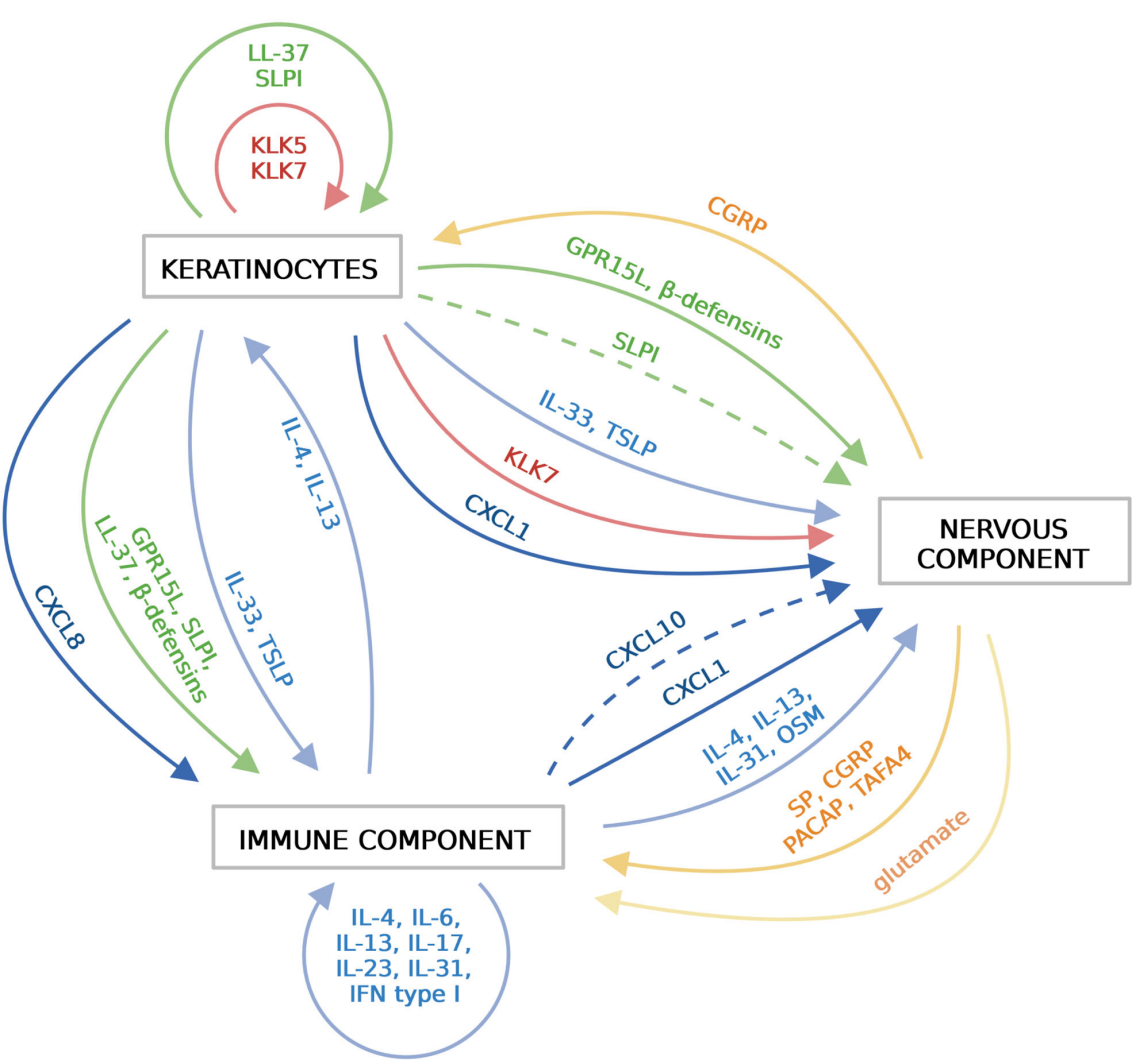
Soluble mediators that functionally link the epidermis, the immune system and the sensory nervous system in the skin. Different components of the skin communicate with each other *via* various soluble mediators, including cytokines (blue), chemokines (dark blue), AMPs (green), proteinases (red), neuropeptides (orange) and nonpeptide neurotransmitters (light orange). Keratinocytes in the epidermis release cytokines, chemokines, AMPs and proteinases, which activate immune cells and/or sensory neurons. Activated immune cells release wide variety of cytokines/chemokines that further amplify immune responses. Sensory neurons sense and respond to local threats or signals from the epidermis or the immune system by releasing neuropeptides. Immune cells and keratinocytes in turn respond to these mediators. Created with BioRender.com.

Cytokines are a biochemically and functionally diverse group of small proteins and peptides that play a key role in cell communication and activation. Among them is a family of chemokines that drive directional cell movement (chemotaxis). Chemokines are classified into four main subfamilies based on a number and position of conserved cysteine residues. The largest two groups include, CC chemokines (the first two out of four cysteine residues are adjacent to each other in this group) and CXC chemokines (which have a single amino acid residue that separates the first two canonical cysteines) (59). In the skin, cytokines and chemokines are best known for their role in inflammation, but accumulating data indicate that keratinocyte- or resident- or skin infiltrating leukocyte-derived cytokines and chemokines can also directly trigger neuronal responses. Thus, cytokines and chemokines can be viewed as epidermal-immune-neuro unit connecting agents in the skin (**Figure 2**).

Alarmin cytokines, such as IL-33 and TSLP that are produced by keratinocytes in response to stress or damage, can activate local immune sentinels as well as recruited leukocytes, including DCs, ILC2, mast cells, basophils or Th2 cells. These cells initiate or propagate allergic inflammation *via* secreting canonical Th2 cytokines such as IL-4, IL-13 and also IL-31 (13). Sensory neurons express the IL-33 receptor ST2 and TSLP receptor TSLPR and respond directly to stimulation by these cytokines with scratching behavior (60, 61). IL-33 is also able to directly activate sensory nerve endings in the skin and act as a mediator of pain (11). As a result of the epidermal barrier breakdown, keratinocytes release various chemokines, including neutrophil-recruiting CXCL8 (IL8) or CXCL1/CXCL2, its ortholog in mouse (12, 16). The role of neutrophils as a neuronal partner in evoking itch was recently shown in the context of experimental AD. In MC903-triggered skin inflammation, neutrophils promoted skin hyperinnervation and upregulation of itch-related signaling mediators such as CXCL10, one of the three chemokine ligands for CXCR3 (16). Building on the observations that CXCL10 was among a small number of genes affected by neutrophil depletion and that treatment with a CXCR3 antagonist attenuated scratching behavior in this model, neutrophils were proposed to promote chronic itch in AD *via* CXCR3-dependent pathways (16).

Apart from keratinocytes, immune cells also release multiple cytokines and chemokines that impact immune cells and neurons in the skin. For instance, in addition to its chemotactic activity, CXCL1 can also activate neurons. CXCL1 is a chemokine produced by macrophages, neutrophils and epithelial cells (62) and its expression can be induced by proinflammatory cytokines such as IL-1, TNF and IL-17. CXCL1 can activate the CXCR2 receptor which, through actin filaments, can activate TRPV1 channels and induce itch (63).

Among the cytokines which have been ‘re-discovered’ in the context of the epidermal-immune-neural connection are IL-4 and IL-13. These cytokines not only drive Th2-dependent inflammation but also act on the epithelial barrier. IL-4 and IL-13 reduce the expression of involucrin and loricrin during keratinocyte differentiation, which may lead to typical AD skin barrier dysfunction (64). Apart from their well-described immune functions, IL-4 and IL-13 also promote itch by acting directly on pruritogenic sensory neurons (65). IL-4 can sensitize neurons to other itch-inducing factors such as histamine. Furthermore, the lack of IL-4Rα specifically on sensory neurons partially protected mice from skin inflammation in an AD mouse model and reduced scratching behavior (65).

Likewise, inflammatory mediators oncostatin M (OSM) and IL-31 affect neuron function in the skin. OSM and IL-31 belong to the IL-6 family of cytokines and share a signaling receptor subunit, OSMβ. The OSM receptor also consists of the gp130 subunit, while the IL-31 receptor consists of IL-31RA (66). OSM is upregulated in AD and psoriatic skin (67). OSM produced by T cells in the skin can directly sensitize itch-selective neurons that express OSMR. Exogenous OSM did not induce significant scratching in mice, while injection of OSM together with histamine augmented scratching when compared to histamine alone. Furthermore, mice with OSMR-deficiency in neurons displayed reduced scratching bouts in a psoriasis model and also attenuated skin inflammation (68). IL-31, typically elevated in AD skin, was the first cytokine discovered to be related to itch (66). Overexpression of IL-31 in mice leads to severe AD symptoms and development of pruritus. IL-31 activated sensory neurons in an AD mouse model and was a primary driver of the itch sensation during disease development (69). However, the specific subunit of IL-31 receptor, IL-31RA, is expressed by other cells present in the skin, such as mast cells, eosinophils and keratinocytes, suggesting that they also may be involved in the activation of the IL-31-dependent immune-neuro circuit. IL-31 and its receptor subunit IL-31RA are promising targets to treat pruritis in various diseases and several drugs are currently under clinical trials. One of them, antibody targeting IL-31RA, nemolizumab, is used for the treatment of pruritus and prurigo and may be approved in the near future (70).

This line of investigation is likely to lead to additional exciting findings on how cutaneous cytokines contribute to neuron function and communication among the epidermal-immune-neuro triad in the skin.

### Antimicrobial peptides and small antimicrobial proteins

Among soluble factors that likely play an important role in connecting the epidermal-immune-neuronal systems are antimicrobial peptides, oligopeptides or small proteins, further collectively referred to as AMPs. Typically, AMPs constitute a diverse group of small peptides in terms of primary amino acid sequence that share several biochemical features. They usually have a net positive charge and form a secondary structure in which hydrophilic and hydrophobic amino acids are segregated spatially (71). This amphipathic design enables AMPs to actively interact with the membranes of microbes, often leading to irreversible membrane damage (72). AMPs can fortify skin defense function not only through killing or restricting the growth of microbes, but also through regulating immune and neuronal responses by directly acting on keratinocytes, immune cells and sensory neurons (**Figure 2**).

Antimicrobial peptide LL-37, a 37-amino acid long cleavage product of cathelicidin protein hCAP18, is one of the best characterized cutaneous AMPs well-known for its proinflammatory properties. Although several cell types can produce LL-37 in the skin (4), keratinocytes are considered the main source of this peptide, especially in the context of skin injury or psoriasis-associated skin trauma (20, 73). By virtue of its ability to form complexes with nucleic acids, LL-37 enables endogenous RNA or DNA to reach their specific intracellular receptors, such as TLR3, TLR7/8 or TLR9 and initiate production of interferon type I (IFNI) or other immune mediators such as IL-6 or TNF. Host derived nucleic acids can be deposited in an extracellular milieu as a result of cell damage (73) or cell activation. The latter involves skin-recruited neutrophils that can release DNA-scaffolded neutrophil extracellular traps (NETs) (74). In epidermal keratinocytes, LL-37 triggers the production of IFNI (interferon-β) in response to double stranded RNA released from dying cells, leading to skin inflammation in the context of skin wounding or psoriasis (73). Psoriasis etiology has also been linked to pDCs, and LL-37 or other AMPs, including β-defensins or secretory leukocyte protease inhibitor (SLPI) (20, 75, 76). PDCs are considered the main producers of IFNI (IFNα) in response to viral nucleic acids. Stimulation of pDCs by endogenous DNA requires coupling of the DNA to LL-37 and delivery of host-DNA to TLR9 in pDCs (20). In this way, LL-37 or other AMPs can drive the production of IFNI in pDCs and unwanted skin inflammation in psoriasis (20, 73).

Among the immune cellular targets of LL-37 in the skin are mast cells (19). LL-37 triggers mast cell degranulation *via* activation of Mas-related G-protein coupled receptor (Mrgpr)X2. MrgprX2 is the human ortholog of the cationic molecule mouse receptor MrgprB2, the same receptor for which neuropeptides such as SP are ligands (45). Mast cells are anatomically and functionally clustered with sensory neurons and implicated in cutaneous inflammation in a variety of chronic inflammatory skin diseases, including AD or rosacea. LL-37 and SP are both linked to mast cell-mediated skin abnormalities associated with rosacea or allergic disorders, respectively (19, 45, 77). Therefore, MrgprB2 may be a receptor that facilitates communication between mast cells and keratinocyte-derived AMPs or sensory nerve fiber-derived neuropeptides relevant to skin pathophysiology.

Another aspect of cross-talk between the epidermis and nervous system involves AMP-mediated control of skin moisture by preventing excessive water loss through the epidermis. SLPI, a superficial keratinocyte-expressed small (~11 kDa) antimicrobial protein is a potential downstream effector of neuronal signaling that tightens the skin barrier by preventing excessive transepidermal water loss (TEWL) (58). Dry skin often acts in the host’s benefit by limiting microbial burden on the surface. However, an increase in TEWL is an indicator of compromised epidermal permeability barrier function of the skin, observed in chronic inflammatory diseases like psoriasis or AD (78). In common with a variety of other AMPs, SLPI was significantly upregulated in psoriatic skin, where it can have an impact on skin inflammation by virtue of its inhibitory activity against neutrophil serine proteases and its immunoregulatory potential (79). In an experimental model of IMQ-induced psoriasis, SLPI was recently found to prevent water loss in uninvolved lateral skin adjacent to the skin site of the IMQ-induced severe skin lesion (58). This SLPI-dependent effect on water retention was attributed to the reflex arc that tightened the barrier *via* SLPI in the lateral skin. In this reflex arc mediated event, sensory neuronal signal initiated at the site of the original skin challenge (in this case the IMQ-treated site) was redirected back to axonal terminals to the adjacent skin area (the lateral skin) (58). Thus, when acting together, sensory neurons and AMPs provide spatially and temporally controlled preventive programs that connect adjacent skin regions. Since skin desiccation often precedes the development of disease flares or worsens their symptoms in psoriasis, these findings suggest that SLPI in cross-talk with the nervous system may facilitate skin hydration, and ameliorate the breakdown of epidermal homeostasis intrinsic to the pathology of psoriasis.

Whereas AMPs such as LL-37 or SLPI link keratinocytes with immune cells or independently with neurons, recent advances also suggest that AMPs can serve to connect keratinocytes with both immune cells and neurons *via* the same family of Mrgpr receptors (14). Building on the observation that Mrgpr receptors have affinity to cationic peptides, several AMPs, including β-defensins and GPR15L were subsequently identified as potential ligands for these receptors (14, 15). Due to its broad antimicrobial activity against bacteria, fungi and viruses, the 9 kDa oligopeptide GPR15L has been categorized as an AMP (80). However, it also regulates skin barrier function by modulating proinflammatory response and late differentiation of keratinocytes (81). Individuals with AD or psoriasis, e. g. pruritus-associated chronic inflammatory disorders, express abnormally high levels of GPR15L in the epidermis (14, 81). Recent findings in an IMQ-based model of psoriasis indicate that GPR15L contributes to psoriasis through its unique capacity to be both vasoactive and prurogenic. In addition to its role as a T cell chemoattractant acting *via* GPR15 (82), GPR15L was also reported to activate sensory neurons in a manner dependent on Mrgpr family member (MrgprA3), and mast cells through stimulation of MrgprX2 and MrgprB2 (14). Subsequent experiments revealed that, although GPR15L stimulated mast cell degranulation, its prurigenic effect was mostly mediated by triggering sensory neuron responses (14). Together, these findings link epidermis-expressed AMPs with sensory neuron-dependent development of itch sensation in psoriasis.

### Neuropeptides

Neuropeptides represent a heterogeneous group of short peptides. They are derived from larger precursors, synthesized in the neuronal cell body and undergoing posttranslational modifications. A single precursor molecule can give rise to a single neuropeptide, multiple different neuropeptides, or multiple copies of the same neuropeptide. In addition, a single neuropeptide precursor can be differentially cleaved to yield different sets of peptides in different cell types. Active neuropeptides are located in dense core granules and transported to the nerve terminal. After being released into the synaptic space, neuropeptides are not recycled and reloaded into synaptic vesicles - neuropeptides must be synthesized *de novo*. In addition to their release at synapses, neuropeptides also act as neurohormones – long-range signaling molecules. Neuropeptides were initially discovered as nervous system regulators, but recent studies have shown that neuropeptides released by nerves innervating the skin can regulate the function of inflammatory cells and affect the function of keratinocytes (83).

Many neuropeptides share key biochemical features with AMPs, such as amino acid composition, cationic charge and secondary structure, suggesting that they might directly control microbial growth (84). The finding that neuropeptides such as SP or human Neuropeptide Y exhibit antimicrobial activity *in vitro* (84, 85) corroborate these predictions. Production of AMPs at the skin surface represents one of the most important preventive strategies to limit skin inhabitant outgrowth and shape the composition of cutaneous microbiota, ultimately restricting virulent microbial strains (4). Notably, recent *in vivo* studies using the model organism *Hydra* demonstrated that the antimicrobial activity of neuropeptides allows them to mold the skin microbiome in innervated skin sites with spatial specificity. These data support the notion that the overall load and composition of the cutaneous microbial community can be controlled by locally distributed neurons expressing specific antimicrobial neuropeptides (86).

Neuropeptides represent one of the main means of communication between neurons and their non-neuronal cellular targets such as immune cells or keratinocytes (**Figure 2**). Recent developments indicate that neuropeptides can be produced by sensory afferents in response to different pathogens or in the context of non-pathogen-induced cutaneous immune and behavioral responses, and directly stimulate DCs, mast cells or macrophages. Cutaneous sensory fibers express a variety of PRRs, including different TLRs. During infection, microbes may act on sensory neurons through these receptors to induce pain (17). Notably, microbial components can also trigger neuronal activation in a manner independent of PRRs. For example, LPS acts directly *via* TRPA1 ion channels on nociceptors causing calcium flux and CGRP release (17). TRPA1 is a chemical nocisensor that responds to a broad range of environmental irritants (87). The identification of another ion channel, TRPV1, as a major determinant of CGRP-mediated Th17 immunity in a model of optogenetic stimulation or *C. albicans*-mediated stimulation of TRPV1+ neurons, suggests a previously unappreciated mechanism of danger detection (43). By triggering pain signals *via* TRPV1 or TRPA1, inflammatory response might be likewise initiated. Interestingly, certain pathogens, including *S. aureus* or *Streptococcus pyogenes* can hijack such CGRP-mediated pathways to limit inflammatory responses (44, 88).

In line with the physical association between DCs or mast cells and neurons in the skin, these cells are often first responders to sensory neuron-derived neuropeptides. Activation of TRPV1+ nociceptors by protease allergens often leads to neuropeptide SP-mediated allergic reaction. For example, the cysteine protease allergen papain was found to activate TRPV1+ afferents to release SP. This neuropeptide bound to MrgprA1 receptors expressed on CD301b+ DCs, promoting their migration to draining lymph nodes (dLNs) where they stimulated differentiation of naïve T cells towards pro-allergic Th2 cells (28). In an experimental contact hypersensitivity (CHS) model, neuropeptide PACAP drove DC migration from the skin to dLNs by upregulation of chemokine receptors CCR7 and CXCR4. This neuropeptide-mediated effect was critical for CHS pathogenesis, since blocking PACAP ameliorated disease (55). In response to house dust mites with cysteine-protease activity, TRPV1+ neurons also promoted mast cell activation *via* release of SP. SP acted on mast cell receptor MrgprB2, leading to their degranulation and subsequent allergic reaction (45).

On the other hand, neuropeptide TAFA4 released by GINIP+ C-fibers was recently reported to play a major role in promoting the tissue-repair function of cutaneous macrophages in a sunburn-like model of skin damage (54). Mice with conditional GINIP+ neuronal ablation demonstrated an impaired macrophage response and higher cutaneous levels of inflammatory cytokines, leading to repair defects and fibrosis. TAFA4 produced by these sensory neurons promoted IL-10 production by dermal macrophages, leading to skin healing after UV damage.

Neuropeptides, such as CGRP and SP can also promote skin inflammation by inducing vasodilation and capillary permeability (89), and play a key role in chronic inflammatory skin disorders. Multiple reports showed elevated levels of neuropeptides in plasma (90) and lesional skin from psoriatic patients (91), and an increased number of CGRP-, VIP- and SP-expressing nerve fibers in psoriatic skin (92). In experimental models, surgical denervation decreased levels of CGRP and SP, and reduced inflammatory infiltrates into lesional skin (93). In the IMQ-induced mouse model of psoriasis, pharmacological ablation of TRPV1+/Nav1.8+ sensory neurons inhibited the release of IL-23 from dermal DC and subsequent IL-17 release by γδ T cells, resulting in reduced recruitment of inflammatory cells (29).

Beyond the direct influence on immune cells, neuropeptides stimulate keratinocyte proliferation and secretion of proinflammatory mediators like IL-1β, Il-6, TNF and NGF. As already mentioned, in AD, cutaneous pruriceptors promote itch-induced scratching behavior. AD lesions are hyperinnervated with increased penetration of nerve endings into the epidermis (94), and can cause keratinocytes hyperproliferation *via* CGRP (95).

### Proteases

Enzymes capable of cleaving peptide bonds in proteins (proteases) are broadly divided into several major families on the basis of their catalytic mechanism (MEROPS-the peptidase database (http://merops.sanger.ac.uk/). These include: cysteine proteases, which have nucleophilic cysteine thiol in a catalytic triad; or serine proteases, which have catalytically essential serine residue at their active site. Endogenous serine proteases in particular, have long been known to play a crucial role in skin homeostasis, cutaneous inflammation and skin-associated behavioral abnormalities. Serine proteases KLKs are responsible for epithelial turnover, by mediating keratinocyte shedding (desquamation) at the skin surface. KLK5 (with trypsin-like target preferences) and KLK7 (equipped with chymotrypsin-like activity) can cleave proteins that structurally join together adjacent keratinocytes in the stratum corneum (6), (**Figure 2**). Hyperactivity of epidermal KLKs that results from mutations in its controlling inhibitor LEKTI is a causative factor for Netherton Syndrome, a skin disorder characterized by stratum corneum detachment and constant AD-like cutaneous manifestations (96). In humans, the KLK family comprises 15 broadly expressed members. Using RNA sequencing to probe the role of KLKs in AD, KLK7 was identified as the most abundant and differentially expressed (compared to normal skin) KLK in AD-affected human epidermis, and in mouse model of AD (10). Subsequent experiments demonstrated that epidermal KLK7 plays a role not only in corneodesmosomal cleavage but also in AD-associated chronic itch without substantially affecting the extent of AD-like inflammation and disease severity (10).

Although certain serine proteases have been implicated in maintaining skin homeostasis as well as contributing to tissue damage and cutaneous inflammatory and behavioral responses, in most cases it remains obscure how the enzymes are mechanistically involved. Recent studies have, however, provided some insight into how serine proteases can directly stimulate cell receptor signaling, such as *via* cleavage-activation of protease activated receptor 2 (PAR2)-dependent pathways.

PARs are a unique family of G protein-coupled receptors that consist of four members. PARs are activated by extracellular and membrane-tethered proteases on PAR+ cells. Serine proteases cleave the extracellular N-terminus of the receptors to reveal tethered peptide ligands. These ligands can then induce cellular signaling *via* binding the cleaved receptor. In the skin environment, some inflammation- pain- or itch-associated responses may be mediated by PAR2 expressed by keratinocytes, mast cells, and/or sensory neurons. For example, KLK5 is PAR2 activator in keratinocytes, and its PAR2-initiated signaling is one of the drivers of TSLP production in the epidermis (96). Likewise, serine proteases that are released from mast cells *via* degranulation have PAR2 activating capacity. In particular, mast cell serine protease tryptase has been associated with deleterious inflammation and pathogenic pain- or itch-related behavioral responses *via* PAR2 activation (97).

Although recent data point to PAR2 as a key target for itch and pain, potentially driven by the release of endogenous proteases from different type of immune cells, the nature of these enzymes remains to be determined (98–100). In addition, due to the ubiquitous expression of PAR2 in the skin that includes keratinocytes, immune cells, and a subset of cutaneous unmyelinated C-fiber sensory afferents that transmit pain (nociceptors) and itch (pruritoceptors) (98–101), it is often difficult to discern whether these behavioral pathological consequences are a result of PAR2-mediated epidermal disruption, or PAR2 activation on leukocytes or sensory neurons. Using PAR2-conditional knockout mice with PAR2 specifically deleted in sensory neurons that express markers that identify itch nociceptors, mechanical and affective pain but not itch was attributed to PAR2 activation in these neurons (99). On the other hand, overexpression of PAR2 in mouse keratinocytes exacerbated scratching in an experimental model of allergen-induced dermatitis, *via* priming of sensory neurons (98). In addition, a causal link between PAR2 and a member of TRP channels, Ca^2+^-permeable cation channel TRPV3 in keratinocytes, was recently reported to mediate pruritus in AD (100). Together, these findings suggest that the PAR2-evoked itch is mediated by keratinocytes and that itch sensation can rely on PAR2-TRP3s coupling in keratinocytes.

## Concluding remarks and further perspectives

The full list of factors that contribute to the organization of the skin barrier by facilitating epidermal-immuno-neuro interactions remains open. Known mediators consist of protein and peptides, lipids, and non-peptide neurotransmitters. As highlighted it this review, new developments that cytokines/chemokines, products of immune cells or keratinocytes, can target sensory neurons, contribute to a more complete picture of the mechanisms underlying skin barrier defense and open new avenues for further research. Moreover, showing that non-PRRs or danger associated receptors but rather pain sensing receptor TRPV1 or chemoreceptor TRPA1 can trigger immune responses *via* neuronal secretion of neuropeptides or other neurotransmitters broadens our understanding of recognition systems and characteristics of soluble mediators that can elicit immune activity.

As with any active field of research, many important questions remain to be elucidated, including local v/s systemic impact of sensory neuron firing in the skin in the context of physiological and pathological processes. Moreover, whereas current research focuses on the skin environment, where dialog between keratinocytes, immune cells and neuron endings is initiated, an important aspect of neuronal signaling takes place in the largely unexplored ganglia or beyond in the central nervous system. For example, the presence and expansion of immune cells not only in the skin but also in DRG or the trigeminal ganglia is also likely to be mechanistically linked to behavioral changes in the context of chronic inflammatory skin diseases.

## Author contributions

EO, KK, PK, AM, NP, MC, PB, BZ, JC performed the literature search. EO, PK, AM, NP, MC, JC drafted the manuscript. JC and BZ edited the manuscript. EO and KK drafted the figures. All authors contributed to the article and approved the submitted version.

## Funding

This research was funded by Polish National Science Center (NCN), grant number UMO-2019/35/B/NZ6/03357.

## Conflict of interest

The authors declare that the research was conducted in the absence of any commercial or financial relationships that could be construed as a potential conflict of interest.

## Publisher’s note

All claims expressed in this article are solely those of the authors and do not necessarily represent those of their affiliated organizations, or those of the publisher, the editors and the reviewers. Any product that may be evaluated in this article, or claim that may be made by its manufacturer, is not guaranteed or endorsed by the publisher.

## Abbreviations

AD, atopic dermatitis; AMPs, antimicrobial proteins and peptides; CGRP, calcitonin gene-related peptide; CHS, contact hypersensitivity; DC, dendritic cell; dDCs, dermal dendritic cells; dLNs, draining lymph nodes; DRG, dorsal root ganglia; GINIP, Gαi-interacting protein; ILC2, group 2 innate lymphoid cells; ILC3, group 3 innate lymphoid cells; IMQ, imiquimod; KLKs, kallikreins; LCs, Langerhans cells; IFNI, type I interferons; MrgprB2, Mas-related G-protein-coupled receptor B2; MrgprD, Mas-related G-protein-coupled receptor D; OSM, oncostatin M; PACAP, pituitary adenylate cyclase-activating polypeptide; PARs, protease activated receptors; PAR2, protease activated receptor 2; pDCs, plasmacytoid dendritic cells; PPRs, pattern-recognition receptors; SCF, stem cell factor; SLPI, secretory leukocyte protease inhibitor; SP, substance P; TEWL, transepidermal water loss; TLRs, Toll-like receptors; TRPA1, transient receptor potential A1; TRPV1, transient receptor potential V1.

## References

[1] Proksch E Brandner JM Jensen JM. The skin: an indispensable barrier. Exp Dermatol (2008) 17:1063–72. doi: 10.1111/j.1600-0625.2008.00786.x 19043850

[2] Byrd AL Belkaid Y Segre JA. The human skin microbiome. Nat Rev Microbiol (2018) 16:143–55. doi: 10.1038/nrmicro.2017.157 29332945

[3] Gallo RL. Human skin is the largest epithelial surface for interaction with microbes. J Invest Dermatol (2017) 137:1213–4. doi: 10.1016/j.jid.2016.11.045 PMC5814118 28395897

[4] Kwiecien K Zegar A Jung J Brzoza P Kwitniewski M Godlewska U. Architecture of antimicrobial skin defense. Cytokine Growth Factor Rev (2019) 49:70–84. doi: 10.1016/j.cytogfr.2019.08.001 31473081

[5] Raymond AA Gonzalez de Peredo A Stella A Ishida-Yamamoto A Bouyssie D Serre G. Lamellar bodies of human epidermis: proteomics characterization by high throughput mass spectrometry and possible involvement of CLIP-170 in their trafficking/secretion. Mol Cell Proteomics (2008) 7:2151–75. doi: 10.1074/mcp.M700334-MCP200 18622020

[6] Caubet C Jonca N Brattsand M Guerrin M Bernard D Schmidt R. Degradation of corneodesmosome proteins by two serine proteases of the kallikrein family, SCTE/KLK5/hK5 and SCCE/KLK7/hK7. J Invest Dermatol (2004) 122:1235–44. doi: 10.1111/j.0022-202X.2004.22512.x 15140227

[7] Kishibe M Bando Y Terayama R Namikawa K Takahashi H Hashimoto Y. Kallikrein 8 is involved in skin desquamation in cooperation with other kallikreins. J Biol Chem (2007) 282:5834–41. doi: 10.1074/jbc.M607998200 17182622

[8] Aberg KM Man MQ Gallo RL Ganz T Crumrine D Brown BE. Co-Regulation and interdependence of the mammalian epidermal permeability and antimicrobial barriers. J Invest Dermatol (2008) 128:917–25. doi: 10.1038/sj.jid.5701099 PMC2671223 17943185

[9] Miller LS. Toll-like receptors in skin. Adv Dermatol (2008) 24:71–87. doi: 10.1016/j.yadr.2008.09.004 19256306 PMC2633625

[10] Guo CJ Mack MR Oetjen LK Trier AM Council ML Pavel AB. Kallikrein 7 promotes atopic dermatitis-associated itch independently of skin inflammation. J Invest Dermatol (2020) 140:1244–52.e4. doi: 10.1016/j.jid.2019.10.022 31883963 PMC7247952

[11] Huang J Gandini MA Chen L M'Dahoma S Stemkowski PL Chung H. Hyperactivity of innate immunity triggers pain *via* TLR2-IL-33-Mediated neuroimmune crosstalk. Cell Rep (2020) 33:108233. doi: 10.1016/j.celrep.2020.108233 33027646

[12] O'Toole EA Mak LL Guitart J Woodley DT Hashimoto T Amagai M. Induction of keratinocyte IL-8 expression and secretion by IgG autoantibodies as a novel mechanism of epidermal neutrophil recruitment in a pemphigus variant. Clin Exp Immunol (2000) 119:217–24. doi: 10.1046/j.1365-2249.2000.01104.x PMC1905536 10606986

[13] Tamari M Ver Heul AM Kim BS. Immunosensation: Neuroimmune cross talk in the skin. Annu Rev Immunol (2021) 39:369–93. doi: 10.1146/annurev-immunol-101719-113805 33561366

[14] Tseng PY Hoon MA. GPR15L is an epithelial inflammation-derived pruritogen. Sci Adv 8 (2022) 8(24):eabm7342. doi: 10.1126/sciadv.abm7342 PMC9200282 35704588

[15] Tseng PY Hoon MA. Specific beta-defensins stimulate pruritus through activation of sensory neurons. J Invest Dermatol (2022) 142:594–602. doi: 10.1016/j.jid.2021.07.178 34480893 PMC9549968

[16] Walsh CM Hill RZ Schwendinger-Schreck J Deguine J Brock EC Kucirek N. Neutrophils promote CXCR3-dependent itch in the development of atopic dermatitis. Elife (2019) 8:e48448. doi: 10.7554/eLife.48448 31631836 PMC6884397

[17] Diogenes A Ferraz CC Akopian AN Henry MA Hargreaves KM. LPS sensitizes TRPV1 *via* activation of TLR4 in trigeminal sensory neurons. J Dent Res (2011) 90:759–64. doi: 10.1177/0022034511400225 21393555

[18] Iwasaki A Medzhitov R. Control of adaptive immunity by the innate immune system. Nat Immunol (2015) 16:343–53. doi: 10.1038/ni.3123 PMC4507498 25789684

[19] Choi JE Werbel T Wang Z Wu CC Yaksh TL Di Nardo A. Botulinum toxin blocks mast cells and prevents rosacea like inflammation. J Dermatol Sci (2019) 93:58–64. doi: 10.1016/j.jdermsci.2018.12.004 30658871 PMC7680644

[20] Lande R Gregorio J Facchinetti V Chatterjee B Wang YH Homey B. Plasmacytoid dendritic cells sense self-DNA coupled with antimicrobial peptide. Nature (2007) 449:564–9. doi: 10.1038/nature06116 17873860

[21] Deckers J Hammad H Hoste E. Langerhans cells: Sensing the environment in health and disease. Front Immunol (2018) 9:93. doi: 10.3389/fimmu.2018.00093 29449841 PMC5799717

[22] Schuler G Steinman RM. Murine epidermal langerhans cells mature into potent immunostimulatory dendritic cells in vitro. J Exp Med (1985) 161:526–46. doi: 10.1084/jem.161.3.526 PMC2187584 3871837

[23] Wang L Bursch LS Kissenpfennig A Malissen B Jameson SC Hogquist KA. Langerin expressing cells promote skin immune responses under defined conditions. J Immunol (2008) 180:4722–7. doi: 10.4049/jimmunol.180.7.4722 18354196

[24] Flacher V Tripp CH Mairhofer DG Steinman RM Stoitzner P Idoyaga J. Murine langerin+ dermal dendritic cells prime CD8+ T cells while langerhans cells induce cross-tolerance. EMBO Mol Med (2014) 6:1191–204. doi: 10.15252/emmm.201303283 PMC4197865 25085878

[25] Shklovskaya E O'Sullivan BJ Ng LG Roediger B Thomas R Weninger W. Langerhans cells are precommitted to immune tolerance induction. Proc Natl Acad Sci USA (2011) 108:18049–54. doi: 10.1073/pnas.1110076108 PMC3207689 22006331

[26] Zhang S Edwards TN Chaudhri VK Wu J Cohen JA Hirai T. Nonpeptidergic neurons suppress mast cells *via* glutamate to maintain skin homeostasis. Cell (2021) 184:2151–66.e16. doi: 10.1016/j.cell.2021.03.002 33765440 PMC8052305

[27] Hosoi J Murphy GF Egan CL Lerner EA Grabbe S Asahina A. Regulation of langerhans cell function by nerves containing calcitonin gene-related peptide. Nature (1993) 363:159–63. doi: 10.1038/363159a0 8483499

[28] Perner C Flayer CH Zhu X Aderhold PA Dewan ZNA Voisin T. Substance p release by sensory neurons triggers dendritic cell migration and initiates the type-2 immune response to allergens. Immunity (2020) 53:1063–77.e7. doi: 10.1016/j.immuni.2020.10.001 33098765 PMC7677179

[29] Riol-Blanco L Ordovas-Montanes J Perro M Naval E Thiriot A Alvarez D. Nociceptive sensory neurons drive interleukin-23-mediated psoriasiform skin inflammation. Nature (2014) 510:157–61. doi: 10.1038/nature13199 PMC4127885 24759321

[30] Yang HW Liu XY Shen ZF Yao W Gong XB Huang HX. An investigation of the distribution and location of mast cells affected by the stiffness of substrates as a mechanical niche. Int J Biol Sci (2018) 14:1142–52. doi: 10.7150/ijbs.26738 PMC6036734 29989093

[31] Wang Z Mascarenhas N Eckmann L Miyamoto Y Sun X Kawakami T. Skin microbiome promotes mast cell maturation by triggering stem cell factor production in keratinocytes. J Allergy Clin Immunol (2017) 139:1205–16.e6. doi: 10.1016/j.jaci.2016.09.019 27746235 PMC5385284

[32] Abreu D Kim BS. Innate immune regulation of dermatitis. Immunol Allergy Clin North Am (2021) 41:347–59. doi: 10.1016/j.iac.2021.04.011 34225893

[33] Clayton K Vallejo AF Davies J Sirvent S Polak ME. Langerhans cells-programmed by the epidermis. Front Immunol (2017) 8:1676. doi: 10.3389/fimmu.2017.01676 29238347 PMC5712534

[34] Yoshiki R Kabashima K Honda T Nakamizo S Sawada Y Sugita K. IL-23 from langerhans cells is required for the development of imiquimod-induced psoriasis-like dermatitis by induction of IL-17A-producing gammadelta T cells. J Invest Dermatol (2014) 134:1912–21. doi: 10.1038/jid.2014.98 24569709

[35] Nestle FO Conrad C Tun-Kyi A Homey B Gombert M Boyman O. Plasmacytoid predendritic cells initiate psoriasis through interferon-alpha production. J Exp Med (2005) 202:135–43. doi: 10.1084/jem.20050500 PMC2212894 15998792

[36] Parisien M Lima LV Dagostino C El-Hachem N Drury GL Grant AV. Acute inflammatory response *via* neutrophil activation protects against the development of chronic pain. Sci Transl Med 14 (2022) 14(644):eabj9954. doi: 10.1126/scitranslmed.abj9954 PMC10317000 35544595

[37] Galli SJ Tsai M Marichal T Tchougounova E Reber LL Pejler G. Approaches for analyzing the roles of mast cells and their proteases in vivo. Adv Immunol (2015) 126:45–127. doi: 10.1016/bs.ai.2014.11.002 25727288 PMC4771191

[38] Mack MR Kim BS. The itch-scratch cycle: A neuroimmune perspective. Trends Immunol (2018) 39:980–91. doi: 10.1016/j.it.2018.10.001 PMC8896504 30471983

[39] Schwendinger-Schreck J Wilson SR Bautista DM. Interactions between keratinocytes and somatosensory neurons in itch. Handb Exp Pharmacol (2015) 226:177–90. doi: 10.1007/978-3-662-44605-8_10 25861780

[40] Yang TB Kim BS. Pruritus in allergy and immunology. J Allergy Clin Immunol (2019) 144:353–60. doi: 10.1016/j.jaci.2019.06.016 PMC6690370 31395149

[41] Meixiong J Anderson M Limjunyawong N Sabbagh MF Hu E Mack MR. Activation of mast-Cell-Expressed mas-related G-Protein-Coupled receptors drives non-histaminergic itch. Immunity (2019) 50:1163–71.e5. doi: 10.1016/j.immuni.2019.03.013 31027996 PMC6531358

[42] Blake KJ Baral P Voisin T Lubkin A Pinho-Ribeiro FA Adams KL. Staphylococcus aureus produces pain through pore-forming toxins and neuronal TRPV1 that is silenced by QX-314. Nat Commun (2018) 9:37. doi: 10.1038/s41467-017-02448-6 29295977 PMC5750211

[43] Cohen JA Edwards TN Liu AW Hirai T Jones MR Wu J. Cutaneous TRPV1(+) neurons trigger protective innate type 17 anticipatory immunity. Cell (2019) 178:919–32.e14. doi: 10.1016/j.cell.2019.06.022 31353219 PMC6788801

[44] Pinho-Ribeiro FA Baddal B Haarsma R O'Seaghdha M Yang NJ Blake KJ. Blocking neuronal signaling to immune cells treats streptococcal invasive infection. Cell (2018) 173:1083–97.e22. doi: 10.1016/j.cell.2018.04.006 29754819 PMC5959783

[45] Serhan N Basso L Sibilano R Petitfils C Meixiong J Bonnart C. House dust mites activate nociceptor-mast cell clusters to drive type 2 skin inflammation. Nat Immunol (2019) 20:1435–43. doi: 10.1038/s41590-019-0493-z PMC6858877 31591569

[46] de Moraes ER Kushmerick C Naves LA. Morphological and functional diversity of first-order somatosensory neurons. Biophys Rev (2017) 9:847–56. doi: 10.1007/s12551-017-0321-3 PMC5662056 28889335

[47] Haberberger RV Barry C Dominguez N Matusica D. Human dorsal root ganglia. Front Cell Neurosci (2019) 13:271. doi: 10.3389/fncel.2019.00271 31293388 PMC6598622

[48] Woolf CJ Ma Q. Nociceptors–noxious stimulus detectors. Neuron (2007) 55:353–64. doi: 10.1016/j.neuron.2007.07.016 17678850

[49] Lowy DB Makker PGS Moalem-Taylor G. Cutaneous neuroimmune interactions in peripheral neuropathic pain states. Front Immunol (2021) 12:660203. doi: 10.3389/fimmu.2021.660203 33912189 PMC8071857

[50] Cranfill SL Luo W. The development of somatosensory neurons: Insights into pain and itch. Curr Top Dev Biol (2021) 142:443–75. doi: 10.1016/bs.ctdb.2020.10.005 PMC8099032 33706924

[51] Roosterman D Goerge T Schneider SW Bunnett NW Steinhoff M. Neuronal control of skin function: the skin as a neuroimmunoendocrine organ. Physiol Rev (2006) 86:1309–79. doi: 10.1152/physrev.00026.2005 17015491

[52] Usoskin D Furlan A Islam S Abdo H Lonnerberg P Lou D. Unbiased classification of sensory neuron types by large-scale single-cell RNA sequencing. Nat Neurosci (2015) 18:145–53. doi: 10.1038/nn.3881 25420068

[53] Ahern CA Payandeh J Bosmans F Chanda B. The hitchhiker's guide to the voltage-gated sodium channel galaxy. J Gen Physiol (2016) 147:1–24. doi: 10.1085/jgp.201511492 26712848 PMC4692491

[54] Hoeffel G Debroas G Roger A Rossignol R Gouilly J Laprie C. Sensory neuron-derived TAFA4 promotes macrophage tissue repair functions. Nature (2021) 594:94–9. doi: 10.1038/s41586-021-03563-7 34012116

[55] Yamamoto Y Otsuka A Ishida Y Wong LS Seidel JA Nonomura Y. Pituitary adenylate cyclase-activating polypeptide promotes cutaneous dendritic cell functions in contact hypersensitivity. J Allergy Clin Immunol (2021) 148:858–66. doi: 10.1016/j.jaci.2021.02.005 33609627

[56] Hsieh ST Lin WM. Modulation of keratinocyte proliferation by skin innervation. J Invest Dermatol (1999) 113:579–86. doi: 10.1046/j.1523-1747.1999.00737.x 10504444

[57] Pavlov VA Chavan SS Tracey KJ. Molecular and functional neuroscience in immunity. Annu Rev Immunol (2018) 36:783–812. doi: 10.1146/annurev-immunol-042617-053158 29677475 PMC6057146

[58] Kwiecinska P Grygier B Morytko A Sanecka-Duin A Majchrzak-Gorecka M Kwitniewski M. Secretory leukocyte protease inhibitor regulates nerve reflex-mediated skin barrier function in psoriasis. J Eur Acad Dermatol Venereol (2022) 36:1266–74. doi: 10.1111/jdv.18065 PMC9546283 35279880

[59] Charo IF Ransohoff RM. The many roles of chemokines and chemokine receptors in inflammation. N Engl J Med (2006) 354:610–21. doi: 10.1056/NEJMra052723 16467548

[60] Liu B Tai Y Achanta S Kaelberer MM Caceres AI Shao X. IL-33/ST2 signaling excites sensory neurons and mediates itch response in a mouse model of poison ivy contact allergy. Proc Natl Acad Sci U S A (2016) 113:E7572–9. doi: 10.1073/pnas.1606608113 PMC5127381 27821781

[61] Wilson SR The L Batia LM Beattie K Katibah GE McClain SP. The epithelial cell-derived atopic dermatitis cytokine TSLP activates neurons to induce itch. Cell (2013) 155:285–95. doi: 10.1016/j.cell.2013.08.057 PMC4041105 24094650

[62] De Filippo K Dudeck A Hasenberg M Nye E van Rooijen N Hartmann K. Mast cell and macrophage chemokines CXCL1/CXCL2 control the early stage of neutrophil recruitment during tissue inflammation. Blood (2013) 121:4930–7. doi: 10.1182/blood-2013-02-486217 23645836

[63] Deftu AF Filippi A Gheorghe RO Ristoiu V. CXCL1 activates TRPV1 *via* gi/o protein and actin filaments. Life Sci (2018) 193:282–91. doi: 10.1016/j.lfs.2017.09.041 28966134

[64] Kim BE Leung DY Boguniewicz M Howell MD. Loricrin and involucrin expression is down-regulated by Th2 cytokines through STAT-6. Clin Immunol (2008) 126:332–7. doi: 10.1016/j.clim.2007.11.006 PMC2275206 18166499

[65] Oetjen LK Mack MR Feng J Whelan TM Niu H Guo CJ. Sensory neurons Co-opt classical immune signaling pathways to mediate chronic itch. Cell (2017) 171:217–28.e13. doi: 10.1016/j.cell.2017.08.006 28890086 PMC5658016

[66] Dillon SR Sprecher C Hammond A Bilsborough J Rosenfeld-Franklin M Presnell SR. Interleukin 31, a cytokine produced by activated T cells, induces dermatitis in mice. Nat Immunol (2004) 5:752–60. doi: 10.1038/ni1084 15184896

[67] Boniface K Diveu C Morel F Pedretti N Froger J Ravon E. Oncostatin m secreted by skin infiltrating T lymphocytes is a potent keratinocyte activator involved in skin inflammation. J Immunol (2007) 178:4615–22. doi: 10.4049/jimmunol.178.7.4615 17372020

[68] Tseng PY Hoon MA. Oncostatin m can sensitize sensory neurons in inflammatory pruritus. Sci Transl Med 13 (2021) 13(619):eabe3037. doi: 10.1126/scitranslmed.abe3037 PMC9595590 34757808

[69] Cevikbas F Wang X Akiyama T Kempkes C Savinko T Antal A. A sensory neuron-expressed IL-31 receptor mediates T helper cell-dependent itch: Involvement of TRPV1 and TRPA1. J Allergy Clin Immunol (2014) 133:448–60. doi: 10.1016/j.jaci.2013.10.048 PMC3960328 24373353

[70] Kabashima K Irie H. Interleukin-31 as a clinical target for pruritus treatment. Front Med (Lausanne) (2021) 8:638325. doi: 10.3389/fmed.2021.638325 33644103 PMC7906974

[71] Brzoza P Godlewska U Borek A Morytko A Zegar A Kwiecinska P. Redox active antimicrobial peptides in controlling growth of microorganisms at body barriers. Antioxidants (Basel) (2021) 10(3):446. doi: 10.3390/antiox10030446 33805777 PMC7998263

[72] Brogden KA. Antimicrobial peptides: pore formers or metabolic inhibitors in bacteria? Nat Rev Microbiol (2005) 3:238–50. doi: 10.1038/nrmicro1098 15703760

[73] Zhang LJ Sen GL Ward NL Johnston A Chun K Chen Y. Antimicrobial peptide LL37 and MAVS signaling drive interferon-beta production by epidermal keratinocytes during skin injury. Immunity (2016) 45:119–30. doi: 10.1016/j.immuni.2016.06.021 PMC4957248 27438769

[74] Zabieglo K Majewski P Majchrzak-Gorecka M Wlodarczyk A Grygier B Zegar A. The inhibitory effect of secretory leukocyte protease inhibitor (SLPI) on formation of neutrophil extracellular traps. J Leukoc Biol (2015) 98:99–106. doi: 10.1189/jlb.4AB1114-543R 25917460 PMC4467168

[75] Lande R Chamilos G Ganguly D Demaria O Frasca L Durr S. Cationic antimicrobial peptides in psoriatic skin cooperate to break innate tolerance to self-DNA. Eur J Immunol (2015) 45:203–13. doi: 10.1002/eji.201344277 25332209

[76] Skrzeczynska-Moncznik J Wlodarczyk A Zabieglo K Kapinska-Mrowiecka M Marewicz E Dubin A. Secretory leukocyte proteinase inhibitor-competent DNA deposits are potent stimulators of plasmacytoid dendritic cells: implication for psoriasis. J Immunol (2012) 189:1611–7. doi: 10.4049/jimmunol.1103293 22786767

[77] Yamasaki K Di Nardo A Bardan A Murakami M Ohtake T Coda A. Increased serine protease activity and cathelicidin promotes skin inflammation in rosacea. Nat Med (2007) 13:975–80. doi: 10.1038/nm1616 17676051

[78] Pons-Guiraud A. Dry skin in dermatology: a complex physiopathology. J Eur Acad Dermatol Venereol (2007) 21 Suppl 2:1–4. doi: 10.1111/j.1468-3083.2007.02379.x 17716284

[79] Majchrzak-Gorecka M Majewski P Grygier B Murzyn K Cichy J. Secretory leukocyte protease inhibitor (SLPI), a multifunctional protein in the host defense response. Cytokine Growth Factor Rev (2016) 28:79–93. doi: 10.1016/j.cytogfr.2015.12.001 26718149

[80] Yang M Tang M Ma X Yang L He J Peng X. AP-57/C10orf99 is a new type of multifunctional antimicrobial peptide. Biochem Biophys Res Commun (2015) 457:347–52. doi: 10.1016/j.bbrc.2014.12.115 25585381

[81] Dainichi T Nakano Y Doi H Nakamizo S Nakajima S Matsumoto R. C10orf99/GPR15L regulates proinflammatory response of keratinocytes and barrier formation of the skin. Front Immunol (2022) 13:825032. doi: 10.3389/fimmu.2022.825032 35273606 PMC8902463

[82] Ocon B Pan J Dinh TT Chen W Ballet R Bscheider M. And cutaneous chemokine ligand for the lymphocyte chemoattractant receptor GPR15. Front Immunol (2017) 8:1111. doi: 10.3389/fimmu.2017.01111 28936214 PMC5594226

[83] Klein Wolterink RGJ Wu GS Chiu IM Veiga-Fernandes H. Neuroimmune interactions in peripheral organs. Annu Rev Neurosci (2022) 45:339–60. doi: 10.1146/annurev-neuro-111020-105359 PMC9436268 35363534

[84] Brogden KA Guthmiller JM Salzet M Zasloff M. The nervous system and innate immunity: the neuropeptide connection. Nat Immunol (2005) 6:558–64. doi: 10.1038/ni1209 15908937

[85] Hansen CJ Burnell KK Brogden KA. Antimicrobial activity of substance p and neuropeptide y against laboratory strains of bacteria and oral microorganisms. J Neuroimmunol (2006) 177:215–8. doi: 10.1016/j.jneuroim.2006.05.011 16808979

[86] Augustin R Schroder K Murillo Rincon AP Fraune S Anton-Erxleben F Herbst EM. A secreted antibacterial neuropeptide shapes the microbiome of hydra. Nat Commun (2017) 8:698. doi: 10.1038/s41467-017-00625-1 28951596 PMC5614986

[87] Koivisto AP Belvisi MG Gaudet R Szallasi A. Advances in TRP channel drug discovery: from target validation to clinical studies. Nat Rev Drug Discov (2022) 21:41–59. doi: 10.1038/s41573-021-00268-4 34526696 PMC8442523

[88] Chiu IM Heesters BA Ghasemlou N Von Hehn CA Zhao F Tran J. Bacteria activate sensory neurons that modulate pain and inflammation. Nature (2013) 501:52–7. doi: 10.1038/nature12479 PMC3773968 23965627

[89] Chiu IM von Hehn CA Woolf CJ. Neurogenic inflammation and the peripheral nervous system in host defense and immunopathology. Nat Neurosci (2012) 15:1063–7. doi: 10.1038/nn.3144 PMC3520068 22837035

[90] Reich A Orda A Wisnicka B Szepietowski JC. Plasma concentration of selected neuropeptides in patients suffering from psoriasis. Exp Dermatol (2007) 16:421–8. doi: 10.1111/j.1600-0625.2007.00544.x 17437485

[91] Remrod C Lonne-Rahm S Nordlind K. Study of substance p and its receptor neurokinin-1 in psoriasis and their relation to chronic stress and pruritus. Arch Dermatol Res (2007) 299:85–91. doi: 10.1007/s00403-007-0745-x 17370082

[92] Chan J Smoller BR Raychauduri SP Jiang WY Farber EM. Intraepidermal nerve fiber expression of calcitonin gene-related peptide, vasoactive intestinal peptide and substance p in psoriasis. Arch Dermatol Res (1997) 289:611–6. doi: 10.1007/s004030050249 9444383

[93] Ostrowski SM Belkadi A Loyd CM Diaconu D Ward NL. Cutaneous denervation of psoriasiform mouse skin improves acanthosis and inflammation in a sensory neuropeptide-dependent manner. J Invest Dermatol (2011) 131:1530–8. doi: 10.1038/jid.2011.60 PMC3116081 21471984

[94] Mollanazar NK Smith PK Yosipovitch G. Mediators of chronic pruritus in atopic dermatitis: Getting the itch out? Clin Rev Allergy Immunol (2016) 51:263–92. doi: 10.1007/s12016-015-8488-5 25931325

[95] Roggenkamp D Kopnick S Stab F Wenck H Schmelz M Neufang G. Epidermal nerve fibers modulate keratinocyte growth *via* neuropeptide signaling in an innervated skin model. J Invest Dermatol (2013) 133:1620–8. doi: 10.1038/jid.2012.464 23283070

[96] Briot A Lacroix M Robin A Steinhoff M Deraison C Hovnanian A. Par2 inactivation inhibits early production of TSLP, but not cutaneous inflammation, in netherton syndrome adult mouse model. J Invest Dermatol (2010) 130:2736–42. doi: 10.1038/jid.2010.233 20703245

[97] Steinhoff M Vergnolle N Young SH Tognetto M Amadesi S Ennes HS. Agonists of proteinase-activated receptor 2 induce inflammation by a neurogenic mechanism. Nat Med (2000) 6:151–8. doi: 10.1038/72247 10655102

[98] Braz JM Dembo T Charruyer A Ghadially R Fassett MS Basbaum AI. Genetic priming of sensory neurons in mice that overexpress PAR2 enhances allergen responsiveness. Proc Natl Acad Sci U S A 118 (2021) 118(8):e2021386118. doi: 10.1073/pnas.2021386118 PMC7923356 33602818

[99] Hassler SN Kume M Mwirigi JM Ahmad A Shiers S Wangzhou A. The cellular basis of protease-activated receptor 2-evoked mechanical and affective pain. JCI Insight 5 (2020) 5(11):e137393. doi: 10.1172/jci.insight.137393 PMC7308051 32352932

[100] Zhao J Munanairi A Liu XY Zhang J Hu L Hu M. PAR2 mediates itch *via* TRPV3 signaling in keratinocytes. J Invest Dermatol (2020) 140:1524–32. doi: 10.1016/j.jid.2020.01.012 PMC7387154 32004565

[101] Amadesi S Nie J Vergnolle N Cottrell GS Grady EF Trevisani M. Protease-activated receptor 2 sensitizes the capsaicin receptor transient receptor potential vanilloid receptor 1 to induce hyperalgesia. J Neurosci (2004) 24:4300–12. doi: 10.1523/JNEUROSCI.5679-03.2004 PMC6729438 15128844

